# Some Haphazard Aphorisms for Epidemiology and Life

**DOI:** 10.3201/eid1601.090030

**Published:** 2010-01

**Authors:** John M. Cowden

**Affiliations:** Health Protection Scotland, Glasgow, Scotland, UK

**Keywords:** Epidemiology, statistics, surveillance, outbreak investigation, research, aphorisms, another dimension

For more than a quarter of a century, I have accumulated from unexpected sources a ragbag of quotations relevant to the practice of epidemiology. I have even coined a few myself. This paper brings many of them together in one place and acknowledges their origins for the amusement and instruction of colleagues. My motives are not entirely altruistic: I do hope to benefit a little from the reflected glory of those I quote—and of course any publication is a bonus.

The collection is necessarily idiosyncratic and personal. It favors quotes from outside public health rather than the better known sayings of experts in the field. Because I am an epidemiologist, it is biased towards epidemiology rather than microbiology or infection control. It pretends to be neither comprehensive nor representative of anything but my own taste, and it obviously cannot include everyone’s favorites. It may be flawed, but I will consider it a success if, like the British Broadcasting Corporation, it “informs, educates, and entertains.”

## Methods

I consulted my memory for quotations of interest to epidemiologists and health protection professionals. Where possible, I confirmed the actual words of quotations and cited their origins. I have categorized the entries subjectively by topic. I have flouted the convention that the Methods section should contain methods exclusively, and that the results and discussion should be confined to their designated sections.

My citations are haphazard. Having tried hard and failed to identify the first quote, I took the advice of W.C. Fields (1880–1946), “If at first you don’t succeed, try, try again. Then quit. There’s no point in being a damn fool about it.” I have accordingly described my sources in as much detail as possible with minimum effort. “If a job’s worth doing, it’s worth doing well enough for the purpose in hand—to do it any better is wasted effort” (Anonymous). My citations, although insufficiently precise for a scientific paper, match or exceed those in most books of quotations. If a reader wants to run a quote to earth, there is enough information to get them started.

I have tried to avoid clichés (though I mention “lies, damned lies, and statistics” in a comment), the knowledge of which confers little cachet on the user, in favor of less widely known and wittier quotes. If there are any clichés, I hope I have been true to the spirit of Samuel Goldwyn (1879–1974), who declared, “We don’t want old clichés. Let’s have some new clichés.”

Most of the quotes speak for themselves, but occasionally I have been unable to refrain from comment. I have not done so systematically.

## Results

### Surveillance

“Being approximately right most of the time is better than being precisely right occasionally.”—Anonymous.

I first saw this saying in a paper by Tom Reilly, an Australian microbiologist. Tom directed me to his source, Richard Platt, an American epidemiologist. Richard acknowledged using it, but denied knowledge of its origin. My search thus involved 3 continents and was ultimately unsuccessful. I have made less effort with subsequent quotes.

“It is the mark of an educated mind to rest satisfied with the degree of precision which the nature of the subject admits and not to seek exactness where only an approximation is possible.”—Aristotle (philosopher, 384–322 bc). “Consistency is more important than accuracy.”—John M. Cowden (epidemiologist, 1953–)

Although this has the virtue of brevity, it is (aptly) not quite true. Both are important, but in surveillance absolute accuracy is unachievable (see Aristotle). Consistency can be achieved and failure to do so is extremely damaging to credibility among the ignorant classes (especially journalists), as has been observed:

“You cannot hope to bribe or twist / Thank God the British journalist / But when you see what he will do unbribed / There’s no occasion to.”—Humbert Wolfe (poet, 1885–1940). “Not everything that counts can be counted, and not everything that can be counted counts.—Albert Einstein (physicist, 1879–1955).”

Good old Albert. Got himself a reputation as one of the cleverest men in history but still managed to get his girlfriend pregnant accidentally. Hence (I paraphrase):

“It just goes to show that God gave man enough blood to run his brains and his genitals, but not at the same time.”—Robin Williams (actor, 1951–). “‘When you are a bit older’ a judge in India once told an eager young British civil servant, ‘you will not quote Indian statistics with that assurance. The government is very keen on statistics—they collect them, add them, raise them to the nth power, take the cube root and prepare wonderful diagrams. But what you must never forget is that every one of those figures comes from the chowty dar, or village watchman, who just puts down what he damn pleases.’”—Josiah Charles Stamp (economist, 1880–1941, in “Some Economic Factors in Modern Life”).

I found this quote, not in the original text, but in Darrell Huff’s “How to Lie with Statistics,” published by Victor Gollancz in 1954. I am indebted to a reviewer for drawing my attention to its source.

### Outbreak Investigation

“You take a mess. You work hard. You clean it up. Life distilled. All the rest is nonsense.”—Harrison Ford (actor, 1942–).

As applicable to outbreak management as it is to life. I’m sure I first saw this in an interview in a British Sunday newspaper, but an online search produced the link www.mensjournal.com/harrison-ford.

“Prejudice is a great time saver. You can form opinions without having to get the facts.”—E.B. White (writer, 1899–1985).

“It is incident to physicians, I am afraid, beyond all other men, to mistake subsequence for consequence.”—Samuel Johnson (lexicographer, 1709–1784).

“If it looks like a duck, and quacks like a duck, we have at least to consider the possibility that we have a small aquatic bird of the family Anatidae on our hands.”—Douglas Adams (science fiction writer, 1952–2001).

“The race is not always to the swift, nor the battle to the strong. ”—Ecclesiastes 9:1.

“The race is not always to the swift, nor the battle to the strong—but that’s the way to bet!”—Damon Runyon (writer, 1884–1946).

Although Runyon gives this excellent advice, an echo of what I was taught as a student—“Common things are commoner”—he is wise enough to know that you should not always bet on the sure thing. Hence:

“One of these days in your travels, a guy is going to come up to you and show you a nice brand-new deck of cards on which the seal is not yet broken, and this guy is going to offer to bet you that he can make the Jack of Spades jump out of the deck and squirt cider in your ear. But, son, do not bet this man, for as sure as you are standing there, you are going to end up with an earful of cider.”—Damon Runyon (in “The Idyll of Miss Sarah Brown”).

And if you wonder how Runyon can hold these two conflicting points of view (i.e., you should bet on what seems most likely, but a bet on a rock-solid certainty will be wrong), see his quote in the Statistics section.

“When you have eliminated the impossible, whatever remains, however improbable, must be the truth.”—Arthur Conan Doyle (author, 1859–1930, in “The Sign of Four”). “It is a capital mistake to theorize before you have all the evidence. It biases the judgment.—Arthur Conan Doyle (in “A Scandal in Bohemia”).”

The latter quote is laudable but impractical advice for practicing public health professionals, who must often make judgments and act (or deliberately refrain from doing so) on the basis of available evidence.

“The man who insists on seeing with perfect clearness before he decides, never decides.”—Henri-Frederic Amiel (philosopher, 1821–1881). “If you choose the most likely option and it turns out to be wrong, it was still the right choice. Presented with the same evidence, you should make the same choice again. If you’re wrong again, re-evaluate your information.”—John M. Cowden. “Life can only be understood backwards, but it must be lived forwards.”—Soren Kierkegaard (philosopher, 1813–1855).

How many times have you intervened on the basis of a hypothesis, subsequently failed to confirm it, and been accused of being precipitate? Or alternatively, waited for the case–control study or microbiology and been accused of unnecessary caution? You can only be sure when to act in retrospect. That is almost worth quotation status by itself!

“Chance favors the prepared mind.”—Louis Pasteur (chemist, 1822–1895). “You won’t be surprised that diseases are innumerable—count the cooks.”—Seneca (philosopher, 4 bc–65 ad). “Circumstantial evidence is not the same as weak evidence. A naked man in your wife’s wardrobe is circumstantial evidence of infidelity.”—John M. Cowden. “All business proceeds on beliefs, or judgments, or probabilities, and not on certainties.”—C.W. Eliot (president of Harvard University, 1834–1926).

### Research

“Of course we don’t know what we’re doing, that’s why it’s called research.”—Albert Einstein.

I have found this in a couple of places online, but not in a primary or even reliable looking secondary source, so, as they say in Scotland, “I hae ma doots.”

“The tragedy of science is the slaying of a beautiful hypothesis by an ugly fact.”—T.H. Huxley (scientist, 1825–1895).

Huxley was an ardent supporter of the theory of evolution and was known as “Darwin’s Bulldog.” His grandson, Aldous (who died on the day Kennedy was shot), was a mystic—which just goes to show that evolution does not necessarily advance in a straight line.

“Belief is no substitute for arithmetic.”—Henry Spencer (computer programmer and Internet pioneer, 19??–)

Although Henry Spencer has a Wikipedia entry, much of which seems to be in a foreign language, I have been unable, in a grueling 15-minute search, to ascertain his date of birth.

“To every complex question there is a simple answer … and it is wrong.”—H.L. Mencken (writer and wit, 1880–1956). “Semmelweis was right—but he died a broken man and was buried in a pauper’s grave.”—John M. Cowden

The example of Ignatz Semmelweis (1818–1865) contradicts Mencken’s view. Before the invention of microbiologists, he proposed and confirmed handwashing as the simple solution to the massive maternal mortality rates at the Vienna General Hospital. He proved, however, that being right does not necessarily make you successful.

“If you can’t explain it simply, you don’t understand it well enough.”—Albert Einstein. “I have yet to see any problem, however complicated, which, when you look at it in the right way, did not become still more complicated.”—P. Anderson (science fiction writer, 1926–2001). “A scrutiny so minute as to bring an object under an untrue angle of vision, is a poorer guide to a man’s judgment than a sweeping glance which sees things in their true proportion.”—A.W. Kinglake (historian, 1809–1891).

I found this last quote in Winston Churchill’s “My Early Life” and nowhere else, so it must be obscure. Churchill attributes it only to “Kinglake,” assuming his reader would know who Kinglake was. Well I did not, so I did an Internet search. He produced an 8-volume history of the Crimean War, which says a lot for his own sense of proportion.

“It’s a scientist’s right to re-examine his theory with each new piece of evidence.”—Eli Talbert (screenwriter, 19??–, in the “CSI: Crime Scene Investigation” episode “$35K O.B.O.”).

Like Sherlock Holmes, Talbert’s character Gil Grissom could furnish many quotations, but this is my favorite. I should have preferred him to say “duty” rather than “right,” but it did not fit the context.

“In great affairs we ought to apply ourselves less to creating chances than to profiting from those that offer.”—F. de la Rochefoucauld (writer, 1613–1680).

The meaning is not immediately apparent, but I think an epidemiologist should take from this that it is better to take advantage of an outbreak and investigate it in detail, rather than design a research project from scratch. It goes well with the previous Pasteur quote. But as an anonymous Frenchman said, “That’s all very well in practice, but will it work in theory?”

“Editors are often accused of fussing over trivialities. What is contained in the paper is said to be of far greater importance than the way in which it is set out. This may be true. Nevertheless carelessness and inconsistency in the preparation of a paper inevitably suggest carelessness and inconsistency in the conduct of the work itself and prejudice the reader against the author. A reasonable compromise is desirable, but accuracy and lucidity must be insisted on.”—Graham Wilson (microbiologist, 1895–1987).

This is from Sir Graham’s “Guidance in Preparing the Typescript of Scientific Papers.” It provides excellent, unpedantic advice (note his use of a preposition with which to end a sentence). Sir Graham was director of the late lamented Public Health Laboratory Service in England. My own dog-eared copy of the guidance was a gift from the best editor ever to have had the dubious pleasure of savaging my work, Professor Norman Noah. The guidance states that requests for reprints “should be addressed to the editor, Monthly Bulletin (Section II), Central Public Health Laboratory, Colindale Avenue London NW9.” Good luck. I am sure that Sir Graham and Professor Noah would have agreed with the next 2 pieces of advice:

“In composing, as a general rule, run your pen through every other word you have written; you have no idea what vigour it will give your style.”—Sydney Smith (clergyman, 1771–1845). “Brevity in writing is the best insurance for its perusal.”—Rudolf Virchow (pathologist and polymath, 1821–1902). “A microbiologist is someone who is happy to use a colleague’s toothbrush, but not his methods.”—Anonymous. “Knowledge is of two kinds: we know a subject ourselves, or we know where we can find information upon it.”—Samuel Johnson.

### Statistics

“To understand God’s thoughts we must study statistics, for these are the measure of His purpose.”—Florence Nightingale (nurse and pioneer in the use and presentation of statistics, 1820–1910). “All models are wrong, but some are useful.”—George Box (statistician, 1919–)

I am indebted to one of this paper’s reviewers for the previous quote.

“Cowden’s first rule of statistics: statistical significance is not the same thing as practical importance. Cowden’s second rule: the more complex the test required to show statistical significance, the less important to an individual the association is likely to be. Lind didn’t need a p-value to show that lime juice prevented scurvy. Cowden’s third rule: the word ‘significant’ without the prefix ‘statistical’ is usually a coward’s way of implying ‘important’ without mathematical evidence.”—John M. Cowden. “People commonly use statistics like a drunk uses a lamppost: for support rather than for illumination.”—Mark Twain (writer and humorist, 1835–1910).

I have seen this last quote attributed to lots of people, but to Mark Twain more often than anyone else. He may have nicked it, however, because he also used Sir Benjamin Disraeli’s (1804–1881) “lies, damned lies, and statistics” in his autobiography without attribution. Maybe he anticipated the following advice:

“Plagiarize! Plagiarize! Remember why God gave you eyes!”—Tom Lehrer (mathematician and satirist, 1928–).

“I came to the conclusion long ago that all life is six to five against.”—Damon Runyon. “If you wait long enough the improbable is bound to happen.”—John M. Cowden. “Random is not the same as haphazard. Random is likely to be representative, haphazard is likely to be biased. Random is much more difficult to achieve.”—John M. Cowden. “Most people have more than the average number of legs.”—Anonymous. “The plural of anecdote is data.”—Raymond Wolfinger (political scientist, 1931–).

I have found the opposite of this last quote, “The plural of anecdote is not data,” attributed to numerous people, though I disagree with it. I also like the anonymous riposte “the plural of datum is not proof.”

“There are three kinds of epidemiologist: those who can count and those who can’t.”—Anonymous (adapted by John M. Cowden).

## Discussion

Winston Churchill (1874–1965) observed, “It is a good thing for an uneducated man to read books of quotations. The quotations, when engraved upon the memory, give you good thoughts.” Four hundred years earlier, Michel Eyquem de Montaigne (1533–1592) said, “I quote others only the better to express myself.” If I’d said that yesterday, I doubt if it would make it into a book of quotations, but Montaigne reinforces Churchill’s point: quotations are often very helpful. I hope those in this collection are also amusing. Having made an effort to identify their origins, I hope that if anyone uses any of them, they reference this paper, which will have the benefit, for me, of enhancing my CV. If, however, they try to pass off a saying as their own, they will be in good company. When the painter James McNeil Whistler (1834–1903) made a witty remark in the hearing of the writer Oscar Wilde (1854–1900) and Wilde said, “I wish I’d said that,” Whistler replied “You will, Oscar. You will.”

